# Tourette syndrome research highlights from 2020

**DOI:** 10.12688/f1000research.75628.2

**Published:** 2022-05-03

**Authors:** Andreas Hartmann, Cyril Atkinson-Clement, Christel Depienne, Kevin Black

**Affiliations:** 1 Department of Neurology, Hôpital de la Pitié-Salpêtrière, Paris, 75013, France; 2 Paris Brain Institute, Hôpital de la Pitié-Salpêtrière, Paris, 75013, France; 3 Institute of Human Genetics,, University Hospital Essen, Essen, 45122, Germany; 4 Department of Psychiatry, Neurology, and Radiology, Washington University School of Medicine, Saint Louis, MO, 63110, USA

**Keywords:** Tics, Tourette syndrome, 2020

## Abstract

We present here research from 2020 relevant to Tourette syndrome (TS). The authors briefly summarize a few reports they consider most important or interesting.

## Introduction

This article is meant to disseminate recent scientific progress on Tourette Syndrome (TS).

## Methods

We searched PubMed during 2020 using the search strategy *(“Tic Disorders”[MeSH] OR Tourette NOT Tourette [AU]) AND 2020[PDAT] NOT 1950:2019[PDAT].* On 15 Feb 2021 this search returned 293 citations. Colleagues also recommended articles, and we attended selected medical conferences (in 2020, mostly online). We selected material for this review subjectively, guided by our judgment of possible future impact on the field.

## Results

### Phenomenology and natural history

Health-related quality of life in 52 adults with TS was explained largely (79% of variance) by four self-report questionnaires measuring severity of depression, anxiety, obsessive-compulsive symptoms, and attention deficit hyperactivity disorder (ADHD), plus the total tic score (TTS) from the Yale Global Tic Severity Scale (YGTSS) ( Isaacs *et al*. 2021). Depressive symptoms were the strongest predictor by far, and TTS was the weakest. This result supports previous studies concluding that attending to non-tic psychiatric symptoms is crucial in providing optimal care for tic patients.

Three German centers drew attention to a spate of patients presenting with unusual, tic-like manifestations that appeared to be driven by a prominent social media influencer ( Müller-Vahl *et al*. 2020b).

### COVID-19

Because of the outbreak of SARS-CoV-2, the medical landscape shifted significantly in 2020. One consequence was the widespread deployment of telemedicine services, which was discussed specifically for TS by Cen *et al*. (2020). The authors address the potential of telemedicine in the fields of psychoeducation, behavioral therapy, pharmacotherapy and programming of DBS devices. Importantly, they emphasize the need for a multidisciplinary telemedicine team to provide comprehensive care for individuals with TS and their families.

At the beginning of the pandemic (spring 2020) worries and concerns were raised on how this could impact people with TS, albeit on a speculative basis ( Robertson *et al*. 2020). Studies published later in the year sought to investigate this. In an Italian cohort, perhaps unsurprisingly, lockdown worsened symptoms in 67% of patients with TS (n = 238), ranging from tics to hyperactivity, rage attacks, obsessions/compulsions and anxiety. Of note, however, about one fifth of patients reported symptom improvement, maybe linked to lessened social exposure ( Conte *et al*. 2020b). Similar observations were made by two further groups ( Graziola *et al*. 2020a; Mataix-Cols *et al*. 2020).

### Epidemiology

Another analysis, of the Swedish population registry, revealed significantly more substance abuse and consequences—including substance-related death—in people with TS ( Virtanen *et al*. 2021). This result was not explained by other psychiatric illness nor by familial effects (assessed by comparison with their siblings without TS). This result adds substance-related death to suicide and accidental deaths previously found by this same group to be elevated in TS, and suggests clinicians should assess substance use in patients and arrange appropriate treatment. From the same group, it was shown that serious transport accidents occur more frequently in people with TS/chronic tic disorders (CTD) but that this is largely explained by comorbid ADHD ( Mataix-Cols *et al*. 2021). Finally, Fernández de la Cruz and Mataix-Cols review the emerging data on higher rates of general medical illness and mortality in TS based on their comprehensive work using the afore-mentioned Swedish population registry ( Fernández and Mataix-Cols 2020).

The question as to whether the prevalence of TS might vary across the globe remains open. Previous studies suggested that TS might be rarer in Sub-Saharian Africa (also Japan) than in North America and Europe, where most epidemiological studies have been conducted so far. Rodin *et al*. challenge this assumption, rather proposing that adequate training and increased public awareness might result in higher recognition of TS in Uganda and elsewhere. This seems to be a sensible proposition, given that TS was considered ultra-rare just a few decades ago in North America and Europe ( Rodin *et al*. 2021).

### Sensory phenomena and premonitory urges

In 61 people with TS, being aware of signals for emerging tics (quality and intensity of premonitory urges) seems to facilitate self-initiated tic suppression, while ruminative tic-associated sensations (defined as “somatosensory hyperawareness” by the authors) did not, which lends support to the use of premonitory urges in behavior therapy of tics ( Matsuda *et al*. 2020).

Sensory hypersensitivity is a frequent feature in patients with TS and should not be associated uniquely or primarily with autism spectrum disorder. In 34 adults with TS, Isaacs *et al*. confirm what had previously been described mostly in youth with TS, and they further show that sensory hypersensitivity is associated with obsessive-compulsive symptom severity ( Isaacs *et al*. 2020). By the same group, a comprehensive review was carried out on this topic with an accent on pathophysiology of sensory processing dysfunction in TS and associated disorders ( Isaacs and Riordan 2020).

A revised version of the PUTS (Premonitory Urges for Tic Disorders Scale - PUTS-R) was proposed by Baumung *et al*., with slight rephrasing compared to the original, and divided into two subscales regarding urge severity and urge quality, the latter being assessed in a subscale trying to define how the patient feels while experiencing the urge ( Baumung *et al*. 2021). Also, the psychometric properties of the original scale were tested in a very large cohort of children (n = 658, subdivided into three age groups: 3 to 7 years, 8 to 10 years, 11 to 16 years) within the European Multicentre Tics in Children Studies (EMTICS) study. Contrary to previous findings, the PUTS also displayed good internal reliability in children under the age of 10. In children 11 years or older, sensory phenomena related to tics and mental phenomena observed in obsessive-compulsive disorder (OCD) could be distinguished. The authors conclude that questionnaires assessing premonitory urges might need to be age-specific ( Openneer *et al*. 2020b).

### Other

Emotional dysregulation is frequently observed in TS and thought to be related to the co-occurence of ADHD, eventually predisposing to explosive outbursts. However, it has so far been mostly assessed in parent-reported questionnaires. Using an observational measure, Hagstrøm *et al*. directly examined children with TS only, ADHD only, TS+ADHD, and controls. Emotional dysregulation was clearly dependent on the presence of ADHD and could not be observed in TS only ( Hagstrøm *et al*. 2021).

A well-written and comprehensive review on one of the foremost therapeutic challenges in TS, i.e., rage attacks. The authors provide a synthesis on rage attacks and anger in the context of TS. Of special note, rage attacks may occur independently of comorbidities (foremost ADHD) in TS, and actually share many common features with tics. The main problem remains treatment where fresh data are lacking, both in the pharmacological and the psychotherapeutic domain, warranting – rather urgently – further research, i.e., randomized controlled trials ( Conte *et al*. 2020a). On this topic, Müller-Vahl *et al*. propose a revised version of their Rage Attack Questionnaire for adults, this becoming the RAQ-R. Testing this new tool in 127 patients with TS (compared to 645 controls), it was found that rage attacks correlated with and ADHD but, interestingly, could also be observed in “TS only” patients ( Müller-Vahl *et al*. 2019).

Aggressive symptoms in youths with TS (n = 47, compared to 32 controls) appear to correlate with the severity of ADHD; overall, there was - somewhat surprisingly - no difference between the TS and the control group. Note, however, that agression and rage attacks may be correlated but are not identical entities ( Benaroya-Milshtein *et al*. 2020).

Two up-to-date and complete reviews of sleep disorders in TS appeared, covering both adults and children ( Jiménez-Jiménez *et al*. 2020; Hibberd *et al*. 2020). Decreased sleep efficiency, decreased percentage of delta sleep, increased nocturnal awakenings and increased PLMS were the most consistent findings in the polysomnographic studies. Sleep disturbances were often associated with comorbid ADHD and/or anxiety.

Self-injurious behavior in TS was comprehensively reviewed by Stafford and Cavanna (2020).

Openneer and colleagues studied a cognitive control task in children with TS, ADHD, neither or both ( Openneer *et al*. 2019). Their results suggest that executive control impairment in TS could be explained by ADHD, not the tic disorder itself.

Kurvits and colleagues present a wonderfully thorough and thoughtful review of disinhibition as a unifying summary of tics and more complex symptoms in TS ( Kurvits *et al*. 2020). They note strengths and weaknesses of this formulation and suggest future studies that may help resolve the debate.

A large study (N = 720) compared compared autistic and compulsive phenomena in children with a clinical diagnosis of either TS or autism spectrum disorder (ASD) ( Gulisano *et al*. 2020). The Children’s Yale-Brown Obsessive Compulsive Scale (CY-BOCS) for ASD measure was abnormal in patients with ASD or TS+ASD, but not in TS patients without ASD. Low and high scores successfully separated ASD from non-ASD, with or without TS, but scores between 1 and 14 on the CY-BOCS ASD did not adequately discriminate the two groups.

### Etiology

**Genetics**

Despite several studies published in 2020, the genetic factors contributing to TS remain largely unknown. These studies are divided into three main approaches: 1) whole exome sequencing (WES); 2) microarrays, which aim to identify rare coding variants or copy number variants with large effects; and 3) association studies that mainly focus on common variants. WES sequencing in a Chinese family with several affected members identified a missense variant in chloride voltage-gated channel 2 ( *CLCN2)* (G161S), which was enriched in a TS cohort ( Yuan *et al*. 2020). Loss-of-function variants in *CLCN2*, encoding chloride channel 2 (CLC-2), cause a leukoencephalopathy with ataxia, a recessive monogenic disorder ( Depienne *et al*. 2013). The association of G161S with TS remain to be confirmed and its functional impact on the channel investigated. Another WES study conducted on 15 Chinese child-parent trios led to the identification of 25 coding *de novo* variants including two that likely disrupt gene function. The same study also identified rare compound heterozygous variants in cadherin EGF LAG seven-pass G-type receptor 3 ( *CELSR3)* in one proband ( Zhao *et al*. 2020b). *CELSR3* encodes the Cadherin EGF LAG seven-pass G-type receptor 3 that may have an important role in cell/cell signaling during nervous system formation and is one of the few genes significantly associated with TS using WES ( Wang *et al*. 2018) ( Willsey *et al*. 2017). A review by Zhang and colleagues highlighted the possible role of variants in ASH1 Like Histone Lysine Methyltransferase ( *ASH1L)* in the etiology of psychiatric disorders including TS, autism spectrum disorders and intellectual disability ( Zhang *et al*. 2021). *ASH1L* encodes a histone-lysine N-methyltransferase specifically trimethylating Lysine 36 of histone H3 forming H3K36me3. *De novo* mutations in this gene mainly cause intellectual disability with autistic traits ( Krumm *et al*. 2015). A single study making use of array comparative genomic hybridization identified a 260-kb duplication on chromosome Xq28 comprising two genes (Vesicle-associated membrane protein 7 - *VAMP7* and Sprouty RTK Signaling Antagonist 3 - *SPRY3*) in a single female patient, inherited from her healthy father. The same duplication has been reported many times as likely benign in Decipher and do not lead to increase expression of the gene in blood, thus association with TS remains speculative. Several association studies focused on candidate genes or candidate regions have suggested possible association of rare or common variants in *CNR1* (cannabinoid receptor 1), Lim Homeobox 6 ( *LHX6),* Inner Mitochondrial Membrane Peptidase Subunit 2 *(IMMP2L)* and Arylacetamide Deacetylase ( *AADAC)* with TS ( Szejko *et al*. 2020) ( Pagliaroli *et al*. 2020). These association studies were performed on case-control populations limited in size and need further replication. Furthermore, a study showed that deletions altering *IMMP2L* (encoding the mitochondrial inner membrane protease subunit 2) do not lead to a substantial mitochondrial dysfunction in fibroblasts of TS subjects, thus questioning the biological relevance of variants in this gene ( Bjerregaard *et al*. 2020). Finally, a recent study showed that socioeconomic status and education have to be taken into account when studying genetic factors involved in TS as these constitute potential confounders limiting the power of current genetic studies studies ( Wendt *et al*. 2021).

**Environmental risk factors**

A monumental and definitive review (for the time being) on the immunology (immune pathways, neuroinflammation, microbiome) of brain development in general and TS in particular was written by one of the foremost specialists in the field ( Martino *et al*. 2020).

A fascinating study was driven by the clinical observation that blinking tics, a common first symptom of tic disorder, are often mistaken for allergic conjunctivitis (AC) by families and primary care physicians. A group in southwest China studied 70 children with provisional tic disorder (PTD) and 70 tic-free controls and found that AC was more than 4 times more common in PTD (74%) than controls (17%) ( Chen *et al*. 2020). They showed it could not all be symptom confusion, as quantitative measures of dry eyes and allergic responses to a skin prick test were also about four times more common in patients with PTD. These results suggest interesting ideas about immune abnormalities leading to tics. We suggest another possibility based on the common patient report that tics developed after a behavior repeated for another reason outlived its provoking stimulus and became chronic, e.g., a child coughed due to an upper airway infection, but then the cough persisted and became a tic. Perhaps sometimes tics develop when underlying host factors turn an externally triggered repeated behavior (like blinking or coughing) into a chronic symptom; previously, a rodent study showed that this two-hit scenario could cause a different movement disorder, dystonia ( Schicatano *et al*. 1997).

### Pathophysiology

**Animal models**

Recanatesi *et al*. offer the interesting observation that sequences of self-initiated movements can be phenomenologically consistent, but their timing may differ substantially from one instance of a sequence to another ( Recanatesi *et al.* 2020, in press). They used a rat model and cortical electrical recordings to inform a hidden Markov model. They showed that a model “produced by reciprocally coupling a high dimensional recurrent network and a low dimensional feedforward one” can produce certain “metastable attractors” with both predictable phenomenological patterns but highly variable timing. Since tics also often occur in stable sequences, a similar model may provide useful, testable hypotheses for how the brain generates such tic sequences.

**Electrophysiology**

Cagle and colleagues reported an interesting study based on LFP recording of both the centromedian thalamic nucleus and the primary motor cortex in four TS patients following bilateral deep brain stimulation surgery ( Cagle *et al*. 2020). They highlighted that beta power (12-30 Hz) was reduced in the primary motor cortex after both a tic and a voluntary action, while low-frequency power (3-10 Hz) was increased after a tic but not after a voluntary movement. The authors identified a tic-specific signal within the centromedian thalamic nucleus that could be a target for developing closed-loop deep brain stimulation.

A vast resting-state EEG study comparing young (7-15 years old) TS patients, chronic tic disorders patients and healthy controls revealed many interesting results ( Naro *et al*. 2020). Among them, they highlighted a disconnection of the fronto-parietal network which could contribute to TS motor symptomatology, while a sensorimotor disconnection was revealed for both TS and chronic tic disorders patients as related to tic severity only. In addition, they identified the dynamic of tics in both groups of patients as follow: (1) for TS patients only, tics are preceded by a gamma (30-70 Hz) frequency activation and a beta2 (20-30 Hz) frequency deactivation in the posterior cingulate cortex and the supplementary motor area; (2) for both, tics onset are associated with alpha (8-13 Hz) and beta (13-30 Hz) deactivation within the sensorimotor areas; (3) for TS patients only, tics are followed by a gamma (30-70 Hz) and beta (13-30 Hz) frequency activation in the left dorsolateral prefrontal cortex, while for chronic tic disorders patients they are followed by a delta (2-4 Hz) and alpha (8-13 Hz) deactivation within the posterior cingulate cortex. Therefore, the dynamic of tics in TS and chronic tic disorders patients are differently disturbed, and the fronto-parietal network disconnection result reinforces the known pathophysiology of TS as related to an impairment of the limbic, paralimbic and cortico-striatal-thalamo-cortical pathways.

Sun and colleagues explored the suppressive effect of the motor system on the sensory system in TS patients ( Sun *et al*. 2020). They used a sham-controlled repetitive Transcranial Magnetic Stimulation (rTMS) protocol (1Hz, 90% of the resting motor threshold) and recorded the somatosensory evoked potentials before and 15 minutes after rTMS. If somatosensory evoked potentials amplitudes were decreased for both TS patients and healthy controls, the decrease was stronger for TS patients. They interpreted this finding as a suppressive effect of the motor-sensory system on the sensory system instead of a sole influence of the motor system, and therefore as TS resulting more from a sensorimotor disorder than a sole motor dysfunction.

### Neuroimaging studies

Rae and colleagues provide a high-quality study of action inhibition in TS comprising 23 adults with TS and 21 healthy controls using the same intentional inhibition task ( Rae *et al*. 2020). Importantly, the authors chose a task that did not directly involve tics, so both groups could participate equally in inhibiting a movement. Several inhibitory regions were more active in TS, especially right inferior frontal gyrus, plus insula and basal ganglia. Even though participants did not move, the primary cortex was more active during the task in the TS group but less active in the control group. Finally, during a task in which participants decide on their own whether or not to move a finger, premonitory sensations correlated with functional connectivity of the pre-supplementary motor area (SMA) region to caudate, globus pallidus and thalamus.

Hippocampal volume was increased both in TS and in 41 children with PTD compared to tic-free control children ( Kim *et al*. 2020). Since the PTD group was studied a mean of only 4 months after tic onset, this difference cannot be due to living with or adapting to tics for years, an advantage over all studies in TS itself. Excitingly, in the PTD group, a larger hippocampus at the initial visit predicted worse tic severity at one-year follow-up, comprising the first predictive biomarker identified for PTD ( Kim *et al*. 2020). Surprisingly, striatal volume at baseline did not predict tic severity at follow-up.

Bhikram and colleagues reported a resting-state functional magnetic resonance imaging (fMRI) study in TS that included 39 TS patients and 20 controls, analyzed using seed-based functional connectivity (fcMRI) methods ( Bhikram *et al*. 2020). The seeds chosen for the analyses included the supplementary motor area and putamen, the orbitofrontal cortex and the caudate and the insula. The TS group showed greater connectivity between the temporal gyri, insula and putamen, and between orbital frontal cortex (OFC) and cingulate cortex. Tic severity correlated with increased connectivity of the putamen with sensorimotor cortex. By contrast, OCD severity correlated with decresae connectivity between SMA and thalamus and between caudate and precuneus. Finally, premonitory urge severity correlated with decreased connectivity between OFC and primary sensorimotor cortext, and inferior frontal gyrus correlated with putamen and insula. Perhaps surprisingly, even though only the first symptom domain reflects actual movement, all three symptom-related networks include sensorimotor regions.

Zapparoli and colleagues reported two interesting studies of the experience of agency, *i.e.,* the appreciation that we intentionally acted, and our actions caused the observed consequences ( Zapparoli *et al*. 2020a, b). These studies involved 25 adults with TS and 25 tic-free control participants, and used an implicit, indirect measure of agency called the intentional binding phenomenon, in which people judge the delay between an action ( *e.g.,* pressing a button) and its effect ( *e.g.,* the turning on of a light) to be shorter with intentional than with passive movement. The earlier report gives results from tic-free participants studied with fMRI and TMS to the pre-SMA. The second report shows that the TS group did not show significant intentional binding or correlation with activity in the network identified in the control group. The more reduced the individual sense of agency, the more severe were the motor symptoms correlations as measured with Yale Global Tic Severity Scale. The authors interpret the results as consistent with impaired action monitoring and an impaired sense of agency in TS, which may contribute to the perception of some people with TS that tics are fully involuntary.

Rage attacks in TS were examined using structural and functional MRI network methods in 55 patients with TS, 47% of whom had intermittent explosive outbursts (IEOs) ( Atkinson-Clement *et al*. 2020c). The group with IEOs (TS+IEO) was compared to the remaining (TS−IEO) group, and showed increased fractional anisotropy in the right SMA and right hippocampus, and decreased mean diffusivity in the left OFC. Those three regions were used as seeds for resting state fcMRI. The TS+IEO group showed lower connectivity within a sensorimotor cortical-basal ganglia network, and altered connectivity among OFC, amygdala, and hippocampus. These results suggest that IEOs are associated in TS with disrupted white matter and associated functional connectivity in circuits related to action selection, emotion regulation, impulse control, and aggression.

Using diffusion tensor imaging and subcortical regions of interest in 15 children suffering from TS and 15 healthy controls, Xia and colleagues found decreased fractional anisotropy (FA, related to white matter myelin integrity, fiber compactness and parallelism) in the left globus pallidus and the left thalamus and an increased apparent diffusion coefficient (ADC, related to the molecular diffusion rate) increased in the right caudate nucleus and the thalamus bilaterally in TS patients ( Xia *et al*. 2020). Moreover, the decreased FA within the left thalamus was related to the YGTSS score.

An MRI surface-based study on an important sample of 60 TS patients and 52 healthy controls was also published this year ( Kong *et al*. 2020). They identified several changes regarding cortical thickness and cortical curvatures, essentially distributed within the frontal, parietal and temporal cortices, but they found no difference regarding local gyrification.

Frequency-specific regional homogeneity (ReHo) was also assessed in children’s patients ( Lou *et al*. 2020). This study revealed an increased ReHo in the left precentral gyrus and a decreased in the right operculum. They also identified ReHo changes in some specific frequency bands within the superior frontal gyrus, the superior parietal gyrus, the anterior cingulate gyrus, the putamen, the superior temporal gyrus and the operculum.

Using graph theory on resting-state fMRI, the study of Openneer *et al.* demonstrated that TS is related to dysfunction within the default mode (for local efficiency and clustering coefficient) and that tic severity is correlated with dysfunction within both the fronto-parietal and the default mode networks without relation with ADHD comorbidity ( Openneer *et al*. 2020a). This suggests an immature topological brain organization specifically related to TS.

### Pharmacological studies

A fascinating study on endocannabinoids was reported using cerebrospinal fluid (CSF) from 20 adults with TS and 19 controls ( Müller-Vahl *et al*. 2020a). The authors measured anandamide (AEA), 2-arachidonoylglycerol (2-AG), palmitoyl ethanolamide (PEA), and arachidonic acid (AA). The key results were that “CSF AEA (p = 0.0018), 2-AG (p = 0.0003), PEA (p = 0.02), and AA (p < 0.0001) were significantly increased in TS compared with controls,” and that “levels of 2-AG correlated with the severity of comorbid ADHD (p < 0.01).” The authors note these differences could relate to compensation for chronic tics, or may be causative.

137 children with CTD were assessed at baseline, during a tic exacerbation, and 2 months later ( Addabbo *et al*. 2020). Serum anti-dopamine-2 receptors (D2R) antibodies were measured. 8% had anti-D2R antibodies during the exacerbation, and 8% of those with 2-month data at 2 months after the exacerbation. The αD2R antibodies were significantly associated with exacerbations, with or without correction for patient characteristics including medication use. These antibodies may possibly worsen tics via antibody receptor blockade. Further research is needed to clarify the causal role. See also the commentary by ( Conceição 2020).

### Clinical and neuropsychological studies

Impaired associative learning was shown in 46 children with TS compared to 46 matched control children who performed the Rutgers Acquired Equivalence Test (face and fish test) ( Eördegh *et al*. 2020). This test includes an acquisition phase (associating two visual stimuli based on feedback of correct *vs.* incorrect), which depends on intact basal ganglia function, and a test phase (retrieval of previous association and generalization to predictable new stimuli), which depends on the hippocampus and medial temporal lobe. The TS group performed worse on the acquisition phase (number of trials and accuracy), regardless of comorbid ADHD, OCD, autism spectrum disorder or medication status. However, they performed normally on retrieval and generalization. However, they performed normally on retrieval and generalization, implicating the basal ganglia. Similarly, two prior studies in TS found abnormal probabilistic classification learning, which also involves the dorsal striatum ( Kéri *et al*. 2002; Marsh *et al*. 2004).

Inhibitory control continues to be a matter of debate in TS. In this topic, a first study explored reactive inhibitory control in adult patients by using a stop-signal task ( Atkinson-Clement *et al*. 2020b). Reactive inhibition was not impaired in all TS patients but only in medicated patients (essentially aripiprazole). In addition, impairment in this group was underpinned by brain structures and functional connectivity of the fronto-temporo-basal ganglia-cerebellar pathway.

A second study from the same group focused on another form of motor impulsivity called “waiting impulsivity” defined as the difficulty to withhold a specific action ( Atkinson-Clement *et al*. 2020a). The authors demonstrated that this form of impulsivity is present in TS patients and correlates with tics severity but is normalised by medication (mainly aripiprazole). In addition, waiting impulsivity in unmedicated TS patients was related to abnormal gray matter intensity in deep limbic structures, and with connectivity between cortical and cerebellar regions.

A third and very interesting study compared automatic and volitional inhibition in 19 adult patients with primary tic disorder in comparison to 15 healthy controls ( Rawji *et al*. 2020). They used a conditional stop-signal task associated with motor cortex TMS to assess reactive volitional inhibition, and a masked priming task to assess proactive automatic inhibition. This opposition is of particular interest since volitional inhibition could be increased to prevent tics to reach the threshold for expression, while automatic inhibition could prevent the initial excitation of the striatal tic focus. The authors found that volitional movement preparation, execution and inhibition are not impaired in patients. Conversely, automatic inhibition was found as impaired in patients which was also correlated to tic severity.

On the same theme of voluntary movements, Mainka *et al*. (2020) published a follow-up study of a previous one on mental chronometry ( Ganos *et al*. 2015). If they found no difference between TS patients and healthy controls on the estimated time of their own voluntary movements and the conscious intention to make a voluntary movement, they identified a linear association between both these variables and the disease duration. The longer the disease duration, the lesser the performances were changed from the data of the first study. For the authors, the chronic tics persistence at adulthood could be associated with developmental impairment of internal premotor processing.

To go further on the assessment of perception-action impairment in TS, members of the same group published an interesting study ( Kleimaker *et al*. 2020). Based on the theory of event coding ( Hommel *et al*. 2001; Hommel 2009), a visual-motor event file task and EEG recording, they found that perception-action binding was increased in Tourette patients and partially correlated with tic frequency. Interestingly, electroencephalography (EEG) results revealed that this process was not solely related to motor and perceptual processes, but also to cognitive processes (i.e., involving the inferior parietal cortex). Based on these results, they conclude that the investigation of perception-action binding in TS is more relevant than the assessment of only motor or perception alone.

The association between real and perceived action in TS was also assessed by using a finger-tapping synchronization task ( Graziola *et al*. 2020b). Interestingly, the authors observed an impaired temporal control in two opposite ways for TS and TS+ADHD patients. The first were “behind the beat”, the second were “ahead of the beat”. This confirmed that TS is related to an impairment of temporal motor control.

This year, two articles also assessed reward evaluation in TS. The first one revealed that adolescents with TS present a higher delay discounting, specifically for large rewards ( Vicario *et al*. 2020). In other words, if they have the choice between a large immediate reward and a larger delayed reward, TS patients are less likely to wait for the larger option than healthy controls. This result is of importance and could contribute to the debate on impulsivity from a more cognitive standpoint. The second one used a reinforcement learning task with various reward probabilities ( Schüller *et al*. 2020). The authors showed that TS patients had lower learning curves than healthy controls, but also that reaction time of the healthy was influenced by the reward amount which was not the case for TS patients. In addition, EEG recording revealed an attenuated P3a (positive fronto-central peaking) modulation was found in TS, which was interpreted as an impaired coding of attention allocation.

### Treatment

**Psychological interventions**

Behavior therapy (BT) is considered to be the first line treatment since publication of the 2019 American Academy of Neurology guidelines, based on controlled randomized trials. In a naturalistic setting (children and adolescents with chronic tics, n = 74) and over a 12 month follow-up period, it could be demonstrated that BT is and remains effective in 75% of patients analyzed, attesting not only to its efficacy but durability over a 12 month period ( Andrén *et al*. 2021).

Internet-based BT programs are investigated by multiple groups to make BT available to a larger number of patients, rendering it thus independent on the availability of trained practitioners and financial considerations in countries where psychotherapy is not reimbursed by social security. Rachamin *et al*. offer preliminary data on internet-based guided self-help comprehensive behavioral intervention for tics (I-CBIT) in 25 youths (passive control group/waiting list, n = 16), and show this approach to be both effective and well received over a 6-month period. Larger trials including an active control group are necessary to confirm these first positive results ( Rachamim *et al*. 2020).

Another way to increase access to BT for tic treatment is group training. The “Tackle your Tics” program is an intensive four-day course based on an enhanced version of ERP (exposure and response prevention). First results in 13 youths offer promising results regarding tic reduction and increased quality of life, with a two month follow-up period. Larger controlled trials with longer follow-up periods are awaited ( Heijerman-Holtgrefe *et al*. 2020).

Still another approach is to train parents as therapists. For that purpose, an instructional video guide (on DVD) based on habit reversal training was developed and applied (n = 33) and compared to in-person training (n = 11) in children (mean age 10 years) ( Singer *et al.* 2020). Home-based, parent-administered habit reversal training was as efficacious for tic reduction as traditional in-person training. However, the drop out rate in the former group was close to 50%, so that the authors advocate regular phone contacts during the DVD treatment course, which squares with other hybrid formats such at BIP TIC (internet-delivered behavior therapy with therapist and parent involvement) ( Andrén *et al.* 2019).

A very small (n = 3) case series described an interesting new BT technique based on attention training to suppress tics: to be followed ( Schaich *et al*. 2020).

So far, BT is usually proposed for children above the age of ten. In this very interesting proof of concept study, Bennett *et al*. test a comprehensive behavior intervention for tics (CBIT) format (“CBIT-Jr.”) for children ages 5-8 (n = 16) and show positive response (tic reduction) and acceptance. Moreover, they monitor these improvements over a one-year period and speculate that early BT might alter the chronic course of tics: this is a very important subject and should be investigated in larger, longitudinal cohorts ( Bennett *et al*. 2020).

Remarkably, CBIT was shown as also normalizing inhibitory control in a specific task of perception-action bindings ( Petruo *et al*. 2020).

**Neurosurgery**

An Italian center reports their experience with anterior globus pallidus internus (GPi) vs. thalamic centromedian-parafascicular complex (Cm-Pf) deep brain stimulation (DBS) for TS ( Servello *et al*. 2020). Forty-one TS patients had DBS in ventro-oralis/centromedian-parafascicular thalamus and 14 had DBS in anteromedial GPi. The authors followed them for 4 years, with follow ups at 6, 12 ,24 and 48 months post-operatively. YGTSS and Y-BOCS improved in both groups (p < .001), but Y-BOCS improved more in the GPi group. Hardware removal was limited to the thalamic DBS group (13/41, *vs.* 0/14 in the GPi group).

The DBS registry (n = 66 bilateral GPi, n = 32 centromedian [Cm] thalamus) has provided additional important information ( Johnson *et al*. 2020). Probabilistic tractography from estimated volumes of tissue activated (VTAs) was used to identify networks correlated with improvement in tics or OCD symptoms. Cleverly, these networks were in turn used as seed regions for “reverse” tractography to identify local “hot spots” and “cold spots.” For GPi targets, connectivity to limbic and associative networks, caudate, thalamus and cerebellum predicted clinical improvement scores. The anteromedial GPi showed higher connectivity to this network, and the extent to which estimated VTA overlapped with this anteromedial region correlated with tic improvement. For Cm targets, connectivity to sensorimotor and parietal-temporal-occipital networks, putamen and cerebellum correlated with tic improvement. The anterior/lateral part of the Cm region was more highly connected to this network. For both sites, connectivity to prefrontal, orbitofrontal and cingulate cortex correlated with OCD improvement. These results suggest that structural connections of focal stimulation sites to specific networks may lead to clinical benefit. Interestingly, the identified networks may differ not only by symptom but also based on the surgical target region.

A German study explained the effect of centromedian thalamic nucleus deep brain stimulation by using probabilistic tractography in 7 TS patients ( Andrade *et al*. 2020). They highlighted that the tic reduction following DBS was related to the degree of stimulation-dependent connectivity between the centromedian thalamic nucleus and the pre-supplementary motor area. Conversely, non-responders had more active fibers that project into non-motor cortical areas.

**Other treatments**

A fascinating report from the University of Nottingham described a potential novel treatment for tics that uses the peripheral nervous system to induce changes in primary sensorimotor cortex ( Morera Maiquez *et al*. 2020). The radical new idea arose from observations associating movement inhibition with 8-14 Hz activity in motor cortex. The authors first showed that rhythmic 12 Hz peripheral stimulation of the median nerve evoked synchronous contralateral EEG activity over primary sensorimotor cortex, whereas arrhythmic stimulation at the same mean rate did not. As hypothesized, median nerve stimulation (MNS) at 12 Hz created small but statistically significant effects on initiation of voluntary movements. Importantly, this stimulation did not meaningfully impair concentration, suggesting that the effect did not operate through simple distraction. Next, they tested 10 Hz MNS in 19 TS patients, and demonstrated using blinded video ratings a significant reduction in tic number and severity during 1-minute stimulation epochs vs 1-minute no-stimulation epochs. Videos accompanying the publication showed dramatic benefit during MNS in some subjects.

Sukhodolsky *et al*. (2020) report intriguing results from a controlled, crossover design, pilot study of real-time fMRI neurofeedback. Tics improved more with real than sham feedback, the improvement was clinically meaningful (3.8-point decline in YGTSS total tic score), and the effect size was 0.59. Surprisingly, however, the two treatment conditions did not differ in the putative mechanism of benefit, namely control over SMA activity. An accompanying commentary is also useful ( Coffey 2020).

The role of the microbiome in the etiology and pathogenesis of various CNS disorders has attracted widespread interest over the past decade, with fecal transplantation being hailed as a potential treatment. Zhao *et al*. (2020a) report in an exploratory trial in children with TS that this approach resulted in a significant tic decrease (>25% on the YGTSS-TTS) in four out of five subjects during the 8-week trial period. However, there was no placebo group and larger, randomized trials are warranted.

Physical exercise is advocated as positive for a plethora of somatic and mental disorders these days, and TS is no exception. Jackson *et al*. propose that aerobic exercise training (kick boxing) decreased tic frequency in young people with TS (n = 18, age 10-20 years), likely through enhancement of cognitive control ( Jackson *et al*. 2020). Interestingly, tic frequency reduction was less in a Tai Chi group, in which cognitive control enhancement was not significantly altered compared to controls. Thus, the type of physical exercise is important, “aerobic” being the key word here ( Jackson *et al*. 2020). In the same vein, but on an observational basis, Pringsheim *et al*. report that in 110 children with TS, less vigorous physical activity indeed correlated with tic severity. This negative correlation could also be found for light exposure and subjective sleep quality ( Pringsheim *et al*. 2021).

Spanish researchers conducted an open trial of a gluten-free diet in 34 TS patients (mostly children) without celiac disease ( Rodrigo *et al*. 2018). After a year, in the 29 patients who did not withdraw due to dietary noncompliance, tics, OCD symptoms and quality of life were all improved substantially compared to baseline. Prospective data on a dietary intervention, as in this study, are greatly needed. However, this study design cannot exclude improvement due to expectation effects or regression to the mean, so a randomized, controlled trial is essential before we can justify adding dietary restrictions to treatment recommendations.

A survey of 90 respondents from 13 countries showed that online support communities offer valuable informational and emotional support to those living with tic disorders/TS and their families, especially in the light of local face-to-face support that is often lacking. However, some disadvantages also became apparent, such as the suggestible nature of tics and being reminded of the challenging nature of tic disorders. Also, some conflict arising within online communities was noted ( Perkins *et al*. 2020).

Complementary and alternative medicine (CAM) blossoms in all of medicine, treatment of TS is no exception. A survey of 110 patients with TS showed that more than two thirds used one or more CAM therapies. The most popular were: stress management, herbal medicine, homeopathy and meditation. 93% reported a decrease in tic frequency and 46% considered CAM more efficient than medication ( Patel *et al*. 2020). Patients reported they often did not mention CAM treatments to their treating physicians, placing the onus on clinicians to ask patients specifically about them. These results also support the crucial need for randomized, controlled trials of any intervention.

## Conclusions

2020 was a rich year in terms of publications and confirms the impression that TS draws increased attraction in the neuroscience community. Hopefully, this will eventually lead to larger collaborative efforts and, especially, longitudinal studies on TS and comorbidities, with a special emphasis on transition from childhood to adulthood.

## Data availability

No data are associated with this article.
