# Supplementary material for: Tourette syndrome research highlights from 2020
Source: F1000Res. 2022 May 3;11:45. Originally published 2022 Jan 13. [Version 2] doi: 10.12688/f1000research.75628.2 (PMC9021667; doi:10.12688/f1000research.75628.2)
Supplement: Peer review history

# Peer review: Reviewer response for version 2

**DOI:** 10.5256/f1000research.121154.r123030

**Published:** 2022-05-11

**Authors:** James F. Leckman

**Affiliations:** 1 Child Psychiatry, Psychiatry, Psychology and Pediatrics, Yale Child Study Center, Yale School of Medicine, New Haven, CT, USA; 2 Department of Psychiatry, Yale University, New Haven, CT, USA

This is a timely and well written review.

 Some possible additions to the review include: 

1. A brief discussion in the Epidemiology section concerning the recently published article in JAMA Neurology - https://jamanetwork.com/journals/jamaneurology/fullarticle/2790403
2. A brief discussion of the impact of social media, e.g., TikTok on individuals with tics (see: https://tourette.org/wp-content/uploads/FINAL-TAA-Functional-tic-like-behaviors_v4.pdf). This could be cited and discussed in the Environmental Risk Factors section.
3. Also, the authors may wish to cite the in the Discussion the soon to be released textbook on Tourette Syndrome by Oxford University Press ( https://www.vitalsource.com/products/tourette-syndrome-davide-martino-james-leckman-v9780197543221?duration=180).

Best wishes,

 James Leckman

Is the review written in accessible language?

Yes

Are all factual statements correct and adequately supported by citations?

Yes

Are the conclusions drawn appropriate in the context of the current research literature?

Yes

Is the topic of the review discussed comprehensively in the context of the current literature?

Yes

Reviewer Expertise:

NA

I confirm that I have read this submission and believe that I have an appropriate level of expertise to confirm that it is of an acceptable scientific standard.

## References

[1] : Association of Tourette Syndrome and Chronic Tic Disorder With Violent Assault and Criminal Convictions. *JAMA Neurol* .2022;79(5) : 10.1001/jamaneurol.2022.0167 459-467 10.1001/jamaneurol.2022.0167 35311941PMC8938899

[2] : Tourette Syndrome. *Oxford University Press* .2022;

# Peer review: Reviewer response for version 1

**DOI:** 10.5256/f1000research.79522.r127168

**Published:** 2022-04-13

**Authors:** Laura Zapparoli

**Affiliations:** 1 Psychology Department and Milan Center for Neuroscience, University of Milano-Bicocca, Milan, Italy; 2 IRCCS Istituto Ortopedico Galeazzi, Milan, Italy

In this article, Hartmann and colleagues provide a thoughtful summary of the most relevant articles published on Gilles de la Tourette Syndrome in 2020. The included papers are adequately chosen and well-presented, with a clear and readable style. In my opinion, it may represent a useful tool for both researchers and clinicians in the GTS and related fields.

 I have only some minor comments.

 p.2: The authors mentioned the deployment of telemedicine services due to COVID-19, which was explicitly discussed for GTS by Cen and colleagues (2020). I would appreciate some details on the domain of telemedicine and GTS.

 p.2: please, define the “ruminative tic-associated sensations”.

 p.3: please, add some details about the two subscales of the Revised Premonitory Urges for Tic Disorders Scale (e.g., what does “urge quality” refer to?).

 p. 3: The authors mentioned two “up-to-date and complete reviews of sleep disorders in GTS”. I would add some details/results of these reviews.

 p. 5: The authors reported a seed-based functional connectivity study by Bhikram et al. (2020): which seeds were considered?

 p. 5: The authors mentioned the study on the sense of agency in GTS by Zapparoli et al. 2020b: I would add that the more reduced was the individual sense of agency, the more severe were the motor symptoms correlations, measured with Yale Global Tic Severity Scale.

 p. 7: I would add some details about the durability of GTS's behavioral therapy.

 p. 8: The acronym “HRT” is not defined in the manuscript.

 p. 8: The authors mentioned a large-scale DBS study using the pallidus internus and the thalamic centromedian-parafascicular complex as target. Can the authors specify the timing of the follow-ups of the YGTSS/YBOCS improvement? 

 In general, I would be consistent in reporting (or not) the statistical details of each study (e.g., p-values).

Is the review written in accessible language?

Yes

Are all factual statements correct and adequately supported by citations?

Yes

Are the conclusions drawn appropriate in the context of the current research literature?

Yes

Is the topic of the review discussed comprehensively in the context of the current literature?

Yes

Reviewer Expertise:

Cognitive Neuroscience, Motor Control, Motor Cognition, Neuroimaging, Movement Disorders.

I confirm that I have read this submission and believe that I have an appropriate level of expertise to confirm that it is of an acceptable scientific standard.

# Response

**Published:** 2022-04-25

**Authors:** Andreas Hartmann

**Affiliations:** Pitié-Salpêtrière Hospital, Paris, France

We thank Dr. Zapparoli for her thoughtful comments and careful review of our manuscript. Concerning the comments raised, they have been addressed to the best of our abilities. Nonetheless, we would like to point out that the aim of this yearly review is not to present studies in detail, but rather point interested readers to papers which they would like to read themselves if their interest has been peaked. However, since this is a multi-author paper, some differences in style and result presentation are inevitable (which includes the presentation of p values, CI etc.), despite our efforts to harmonize the paper. Also, apart from original studies, summarizing reviews is a complicated affair since they already represent summaries, but we tried to highlight the salient points.

# Peer review: Reviewer response for version 1

**DOI:** 10.5256/f1000research.79522.r119803

**Published:** 2022-02-08

**Authors:** Lorena Fernández de la Cruz

**Affiliations:** 1 Department of Clinical Neuroscience, Centre for Psychiatry Research, Karolinska Institutet, Stockholm, Sweden; 2 Stockholm Health Care Services, Stockholm County Council, Stockholm, Sweden

This manuscript presents a scoping review of the published literature on the topic of Tourette Syndrome (TS) during 2020. The search is not designed to be exhaustive (e.g., only one database is exploded) and the highlighted articles, presented in narrative form, have been selected based on the subjective judgement of the authors. This, however, works very well as a text-book summary for those in and outside the field of TS, turning it into a compulsory yearly read. I commend and congratulate the authors for doing this for yet another consecutive year.

 My only minor criticism (or rather a wish) is that this 2020 review could have appeared earlier. I sincerely appreciate the effort and time behind this manuscript, but the more than 1 year lapse between the review content and the current date makes some (not all) sections appear outdated when I read them now (e.g., functional tics in relation to the Covid-19 pandemic, and some of the treatment parts). On the other hand, some time lag is to be expected, and it is good to see the quick development of the field.

 I have some minor suggestions for improvement of the text, mainly referred to typos, if the authors believe that they are helpful:

- In the Methods section, the authors link the output of their PubMed search. When clicking the link, this search returns 293 citations. However, in the text, the authors write that this number is 292. Please amend.
- In the Results>Epidemiology section, CTD is incorrectly defined as “connective tissue disease”. Please change this to “chronic tic disorder”.
- Results>Other section: I would appreciate if the authors could add a more informative summary of the systematic review by Conte et al. about rage attacks (it currently reads: “many questions remain open and much work needs to be done”).
- Please note that the two references by Conte et al. 2020a and 2020b have been cited in the wrong order. Conte et al 2020b (rage attacks in the Reference list) is cited in the Covid section, while Conte et al 2020a (Covid-19 survey in the Reference list) is cited in the rage attacks paragraph.  
- Results>Other: “Two up-to-date and complete review of…” Please change “review” to “reviews”.
- Pathophysiology>Electrophysiology section: I believe the following sentence does not read well: “They concluded to the identification of a tics’ specific signal within the centromedian thalamic nucleus which could be a target for developing closed-loop deep brain stimulation.” If I understand the conclusion correctly, it should probably say: “The authors identified a tics’ specific signal within the centromedian thalamic nucleus could be a target for developing closed-loop deep brain stimulation.”, or similar wording.
- Clinical and neuropsychological studies section: Please revise the following sentence: “However, they performed normally on retrieval and generalization. Compare two prior studies showing that people with TS have abnormal probabilistic classification learning, which also involves the dorsal striatum.”
- Treatment>Psychological interventions: In the paragraph summarizing the paper by Singer et al., BIP TIC is mentioned, but it is not explained what it is and no reference for the reader is given (the reference by Singer is cited at the end of the sentence but this paper does not seem to mention BIP TIC).
- Other treatments section: The name of the authors Sukhodolsky et al. should be outside the parenthesis.

Is the review written in accessible language?

Yes

Are all factual statements correct and adequately supported by citations?

Yes

Are the conclusions drawn appropriate in the context of the current research literature?

Yes

Is the topic of the review discussed comprehensively in the context of the current literature?

Yes

Reviewer Expertise:

Tourette syndrome, OCD, epidemiology, CBT

I confirm that I have read this submission and believe that I have an appropriate level of expertise to confirm that it is of an acceptable scientific standard.

# Response

**Published:** 2022-02-08

**Authors:** Kevin J Black

**Affiliations:** Department of Psychiatry, Washington University in St Louis, USA

We thank Dr. Fernández de la Cruz for her thoughtful and thorough review. We will address her helpful suggestions after receiving those of a second reviewer.

# Response

**Published:** 2022-02-10

**Authors:** Andreas Hartmann

**Affiliations:** Pitié-Salpêtrière Hospital, Paris, France

We thank Dr. Fernández de la Cruz for her thoughtful comments and careful review of our manuscript. To begin with, we fully agree that submission this year occurred much too late, for which we would like to present our apologies. Frankly speaking, 2021 was a bit of an *annus horribilis* for us, with delays due to COVID-related, family and authorship matters. Usually, these reviews appear in the first half of the upcoming year, as will be the case again in 2022 for the 2021 review. As to the comments raised, we agree with all of them, and they were accordingly addressed.
